# Dr. James West Roosevelt

**Published:** 1896-05

**Authors:** 


### Obituary.

Dr. James West Roosevelt, of New York, died suddenly of pneumonia on Friday, April 10, 1896, in the thirty-eighth year of his age. Not often has the profession been so shocked through the loss of one of its members as it was in this instance. He developed the first symptoms on Thursday morning; the disease advanced with astonishing rapidity, edema of the lungs supervened and eighteen hours from the initiative chill Dr. Roosevelt was dead. This is a singular exhibition of the mysterious and alarming rapidity with which pneumonia may progress to fatality. Dr. Roosevelt was an active member of several medical societies and social clubs. For a number of years Dr. Roosevelt devoted much of his time to investigations of diseases of the lungs, and a little over a year ago he organised the Seton hospital for consumptives at Spuyten Duyvil, in which institution he took a great interest and at his own expense fitted up its very complete pathological laboratory. Dr. Roosevelt was fast achieving fame as a clinician and investigator, and for some years had been attending physician at the Roosevelt and Bellevue hospitals. He was a frequent contributor to medical and lay magazines and was a ready and forceful speaker in medical society discussions. Dr. Roosevelt possessed great amiability of character and his attached friends were many and strong. His widow survives him with three children.

